# Supplementary material for: Looking at the Full Picture: Utilizing Topic Modeling to Determine Disease-Associated Microbiome Communities
Source: bioRxiv. 2023 Jul 25:2023.07.21.549984. Preprint. [Version 1] doi: 10.1101/2023.07.21.549984 (PMC10401927; doi:10.1101/2023.07.21.549984)
Supplement: 1 [file NIHPP2023.07.21.549984V1-supplement-1.pdf]

bioRxiv preprint doi: https://doi.org/10.1101/2023.07.21.549984; this version posted July 25, 2023. The copyright holder for this preprint (which was not certified by peer review) is the author/funder. This article is a US Government work. It is not subject to copyright under 17 USC 105 and is also made available for use under a CC0 license.

Supporting Information

Supplementary Figure 1: The ideal topic number for each dataset is at the minimum value that both metrics generally reach. For the Chen dataset 33 topics was ideal and for Yadav 27 topics was ideal. On average, 30 topics is ideal for these datasets and was selected.

Supplementary Figure 2: a) The 10 of 30 significant topics found in the Chen dataset. b) The 4 of 30 significant topics found in the Yadav dataset. The left-hand side contains the probability of the genus being assigned to the topic. The right-hand side contains the abundance comparison between MS and HC of the highly assigned bacteria. Bolded genera are significant. Positive values indicate a higher abundance in MS compared to HC.

Supplementary Table 1: Demographics for all datasets.
